# Examining the Impact of Digital Components Across Different Phases of Treatment in a Blended Care Cognitive Behavioral Therapy Intervention for Depression and Anxiety: Pragmatic Retrospective Study

**DOI:** 10.2196/33452

**Published:** 2021-12-17

**Authors:** Monica S Wu, Robert E Wickham, Shih-Yin Chen, Connie Chen, Anita Lungu

**Affiliations:** 1 Lyra Health Burlingame, CA United States; 2 Department of Psychological Sciences Northern Arizona University Flagstaff, AZ United States; 3 Department of Psychology Palo Alto University Palo Alto, CA United States

**Keywords:** blended care, cognitive-behavioral therapy, depression, anxiety, digital, phase, mental health, digital health, digital therapy

## Abstract

**Background:**

Depression and anxiety incur significant personal and societal costs. Effective psychotherapies exist, such as cognitive behavioral therapy (CBT); however, timely access to quality care is limited by myriad barriers. Blended care therapy models incorporate traditional face-to-face therapy with scalable, digital components of care, expanding the reach of evidence-based care.

**Objective:**

The aim of this study is to determine the effectiveness of a blended care CBT program (BC-CBT) in real-world settings and examine the unique impacts of the (1) digital components of care (video lessons and digital exercises) and (2) phase of treatment (early versus late) in decreasing symptoms of anxiety and depression.

**Methods:**

This retrospective cohort analysis included 3401 US-based individuals enrolled in a BC-CBT program, who presented with clinical levels of depression and/or anxiety. The treatment program consisted of regular therapy sessions augmented by clinician-assigned digital video lessons and exercises. A growth curve model incorporating time-varying covariates examined the relationship between engagement with BCT components (ie, therapy sessions, digital video lessons, and digital exercises) during the early (weeks 0-7) and late (weeks 8-15) phases of treatment, and weekly symptom reports on depression and anxiety measures.

**Results:**

On average, a significant decline in depression and anxiety symptoms was observed during the initial weeks of treatment (*P*<.001), with a continued, though slower, decline over subsequent weeks (*P*<.001). Each session completed was associated with significant decreases in anxiety (b=–0.72) and depression (b=–0.83) in the early phase, as well as in the late phase (anxiety, b=–0.47; depression, b=–0.27). Significant decreases in anxiety (b=–0.15) and depression (b=–0.12) were observed for time spent on video lessons (measured in 10-minute intervals) in the early phase of treatment. Engaging with exercises was associated with statistically significant increases in anxiety symptoms (b=0.03) during the early phase of treatment. However, sensitivity analyses examining the effects of exercises in isolation revealed significant decreases in anxiety (b=–0.05) in the early phase, suggesting a potential suppression effect in the larger model.

**Conclusions:**

Using a retrospective cohort design, therapy sessions and digital video lessons were uniquely predictive of improvements in depression and anxiety symptoms, and their effects were modulated based on the phase of treatment (early vs late). Future research should investigate whether other treatment variables, such as therapeutic alliance or familiarity with technology, are related to differential effects on various components of care.

## Introduction

Anxiety and depression are leading causes of disability worldwide, costing the global economy US $1 trillion each year due to lost productivity [1]. Given these significant societal and personal burdens [2,3], access to timely, quality care is imperative. Cognitive behavioral therapy (CBT) is considered the gold-standard psychotherapy for anxiety and depression and has robust empirical support [4]. However, obtaining access to CBT is hindered by various barriers, including the costs of therapy, long waiting lists, paucity of qualified mental health professionals, and lack of mental health resources (especially in rural areas) [5].

To overcome these barriers, teletherapy and internet-based CBT (iCBT) are promising ways to bridge the gap in accessing quality mental health care through technology. Video-based teletherapy allows treatment sessions to occur over a videoconferencing platform in real time, and it has shown similarly robust treatment outcomes when compared to in-person CBT for depression and anxiety [6]. Teletherapy facilitates access to therapy regardless of travel limitations or lack of resources in certain geographic locations, expanding the reach of evidence-based care [7].

Similarly, the use of iCBT permits remote access to evidence-based therapy materials, delivered through various digital media (text, audio, video, interactive, and gamified formats) [8]. Treatment materials are deliverable in a scalable and asynchronous manner, allowing for broader dissemination and convenient access to evidence-based therapy content. Targeting anxiety and depression through iCBT has shown significant decreases in symptom severity [9,10], although greater treatment effects are observed when iCBT is performed with therapist support versus without [11]. Indeed, difficulties with retention and smaller treatment effects arise without therapist guidance [12], especially when implemented in real word conditions [13], highlighting the potency of therapist involvement. Combining teletherapy and iCBT harnesses the advantages of both modalities of treatment, lending support for a blended care model of therapy.

Blended care therapy combines traditional face-to-face therapy with digital components of care that cover key therapeutic concepts and skills. Through therapist involvement in regular therapy sessions, clients are able to receive personalized care, discuss therapy content in depth, and be held accountable for homework completion. The digital materials help maintain fidelity to evidence-based practices, reinforce key concepts and skills outside of session, and facilitate rapid dissemination of therapeutic content in a scalable manner [14]. Blended care CBT (BC-CBT) integrates the benefits of both modalities and is a potentially cost-effective manner of delivering treatment [15]. Additionally, CBT delivered in a blended care format has preliminarily demonstrated significant decreases in depression and anxiety symptoms, highlighting its effectiveness in real-world settings [16].

Despite these benefits, there is limited research on the effectiveness of BC-CBT at scale, and whether the different digital components of care (eg, video lessons, digital exercises) have varied impacts on treatment outcome. Teasing apart the unique contributions of the digital components will help better inform which components are most potent in decreasing symptom severity. Additionally, the timing of when various care components are assigned could be an influential factor. Given that symptom changes in psychotherapy often do not follow a linear path [17], it is prudent to consider how different treatment components may contribute to symptom reduction in the early versus late phases of therapy. As various treatment components and process variables may be more or less potent at different times [18], examining these variables in a time-sensitive manner will allow for increased precision and personalization of care to optimize treatment outcomes. Early response and treatment gains at the beginning of therapy have received particular attention [19], as clients who experience early response to treatment tend to achieve better posttreatment outcomes for anxiety and depression [20]. The later phase of therapy is critically important as well, as it typically focuses on translating the therapeutic concepts and skills (learned in the earlier phase of therapy) into day-to-day practice, which is crucial for generalizing these skills [21]. Indeed, facing challenging situations and implementing regular out-of-session skills practices lead to long-term decreases in symptom severity and strengthen the durability of treatment gains [22,23]. Taken together, these findings highlight the importance of examining symptom changes in the early versus late phases of therapy, given the unique impact of each phase of therapy.

Ultimately, a gap remains in our understanding of the unique impact of the digital components of care in BC-CBT, as well as how the phase of treatment may impact treatment outcomes. Consequently, this study aims to evaluate the unique impact of treatment sessions, digital video lessons, and digital exercises in a blended care CBT intervention for depression and anxiety in real-world settings, examined across early versus late phases of treatment. These data are intended to better inform how to optimize care by customizing the different treatment components within a BC-CBT program and the timing of their assignment.

## Methods

### Study Design and Procedures

This study employed a retrospective cohort design, analyzing data collected as part of routine quality control for the BC-CBT program at Lyra Health. Lyra Health is partnered with Lyra Clinical Associates to provide therapeutic services to clients through the BC-CBT program. Clients are employees (or their dependents) of companies that offer mental health benefits through Lyra. Individuals were informed of their behavioral health benefits through their employers, and they could receive care with no cost to them using a set number of Employee Assistance Program sessions or, for certain employers, could receive care through their employer-sponsored health plan. Individuals who accessed the services through their employer-sponsored health plan may have been required to pay a copay, coinsurance, or applicable deductible for sessions through the health plan. Interested clients registered on the web and completed a triage flow to indicate their presenting issues, preference for modality of care, and brief treatment history. If they were appropriate for the BC-CBT program, they were eligible to select the program on the web and book care with a provider. All clinical activities (therapy sessions, questionnaires, digital activities) were conducted over a secure, Health Insurance Portability and Accountability Act (HIPAA)–compliant online platform developed by Lyra Health. Clients were prompted to complete standardized measures of anxiety and depression weekly for the duration of care. Clients were assigned digital activities by their providers, and engagement with and completion of these activities were tracked through the online platform. This retrospective analysis of deidentified data collected during therapy was determined to not be human subjects research by the Palo Alto University institutional review board.

### Participants and Data Inclusion

Participants included in the study were individuals who started BC-CBT treatment between August 1, 2019, and May 3, 2021. Participants were required to have scored above the clinical cutoff for either the Generalized Anxiety Disorder-7 (GAD-7; score ≥8) or the Patient Health Questionnaire-9 (PHQ-9; score ≥10) on a valid baseline assessment (N=3674). To meet inclusion criteria for the BC-CBT program, clients were required to be 18 years of age or older and to be willing to see a provider via video. Clients were excluded from the BC-CBT program if they reported active suicidality, self-harm, or homicidality, or if they had a current diagnosis of severe alcohol/substance use disorder(s), psychiatric disorder with psychotic features that are not stabilized by medications, or unstable bipolar disorder.

**Figure 1:**
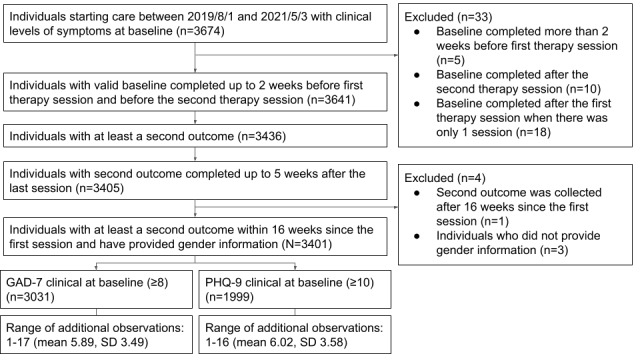
Participant flow. GAD-7: Generalized Anxiety Disorder-7; PHQ-9: Patient Health Questionnaire-9.

Baseline assessments were considered invalid if they were collected more than 2 weeks prior to the first therapy session or after the second therapy session with the provider. Additionally, participants were considered to be missing a valid second assessment if no additional assessment beyond the baseline was completed within 5 weeks after the last therapy session. Assessments were also excluded if they were collected more than 16 weeks after the first therapy session, which represents the mean plus 1 standard deviation of the treatment duration for the sample. Please see Figure 1 for a comprehensive diagram of the participant flow.

### Blended Care Therapy Program

The BC-CBT program included both regular face-to-face therapy sessions with the providers and digital components of care. Providers conducted therapy sessions via video-based teletherapy, generally starting with weekly sessions and gradually titrating to biweekly sessions. Providers assigned digital activities through the platform, and these activities were personalized based on the client’s presenting issues. Completion of assigned digital activities could be monitored in real time via the platform by providers, who were encouraged to send secure messages to their client to remind and/or reinforce completion of assignments. More details regarding the BC-CBT online platform can be accessed via Lungu et al (2020) [16].

#### Therapy Sessions

A short-term, goal-based model was employed, and providers used evidence-based care based on principles from CBT, dialectical behavior therapy (DBT), and acceptance and commitment therapy (ACT). Providers were extensively vetted through a rigorous multistep interview process, and received intensive training in CBT and the proprietary online platform. Ongoing quality assurance was conducted via random session video reviews, regular consultation meetings, and continuing education presentations.

#### Digital Activities

The digital components of the BCT program included *digital video lessons* and *digital exercises*. *Digital video lessons* employ a storytelling approach, which has been shown to be helpful for enhancing relatability and normalization of the presenting issues [24]. These videos follow a character going through therapy, presenting key CBT-based concepts and skills. Example topics include managing emotions, addressing thinking traps, challenging avoidance, and mindful awareness. The video lessons are ~8 minutes in length on average, and a brief quiz is administered at the end of each video lesson to check for understanding.

*Digital exercises* are analogous to paper logs or handouts that facilitate practice of therapy skills. They include awareness-building exercises (eg, thought record) or practice-oriented exercises (eg, exposure practice, behavioral activation, distress tolerance). Providers assign digital exercises to the clients based on their presenting needs, and clients are able to complete them asynchronously at their convenience. Exercise responses can be viewed in real time through the platform by both the provider and the client, and the provider is able to comment on the exercise to reinforce completion and troubleshoot any issues that arise.

### Measures

#### Demographics

Information regarding client demographics (eg, the client’s sex, ethnicity, and birthdate) was collected through the initial intake form completed on the online platform. Minority status was defined by the selection of a non-White group, including American Indian or Alaska Native, Asian or Pacific Islander, Black or African American, Hispanic or Latino, Native Hawaiian or Other Pacific Islander, “multiple,” or “other.”

#### PHQ-9 Score

The Patient Health Questionnaire 9 (PHQ-9) is a 9-item self-report questionnaire that assesses the presence and severity of depressive symptoms in the past week. A cutoff score of ≥10 on the PHQ-9 has been utilized to indicate a likely diagnosis of major depression [25].

#### GAD-7 Score

The GAD-7 is a 7-item self-report questionnaire that assesses the presence and severity of anxiety symptoms in the past week [26]. A cutoff score on the GAD-7 of ≥8 has been used to indicate a likely diagnosis of generalized anxiety disorder [27].

Both symptom severity measures have undergone extensive validation in numerous clinical trials, demonstrating strong psychometric properties as supported by high validity, reliability, and treatment sensitivity [28].

#### Digital Activity Engagement

The online platform records whether or not the client has engaged with the digital video lesson or the digital exercise. The time spent watching digital video lessons is recorded in the system and was coded in 10-minute intervals for the purposes of this study. The completion of digital exercises is logged in the system each time it is submitted by the client.

### Data Analyses

A mixed effects modeling approach to growth curve analysis was employed, which accommodates individually varying time intervals for outcome responses while accounting for missing data under the conditional missing at random (MAR) assumption. This approach also allows for the incorporation of time-invariant covariates (TICs) to model the impact of stable client-level attributes on individual trajectories, as well as time-varying covariates (TVCs) to model the predictive utility of attributes that vary across clients during the course of treatment. Analyses were conducted with SAS PROC MIXED, version 9.4, using restricted maximum likelihood estimation [29]. Because the focus of therapy is tailored and different outcomes may be observed based on the primary presenting issue [30], analyses were conducted separately for clients meeting the threshold for elevated anxiety (GAD-7 ≥8) or depression (PHQ-9 ≥10). If a client exceeded the thresholds for both anxiety and depression, they were included in both analyses.

Following cleaning and validation, a series of hierarchically structured individual growth curve models were examined. The initial analysis (Model 1) for each outcome (PHQ-9, GAD-7) featured a conditional model characterized by fixed effects corresponding to a second-order (quadratic) trajectory, along with client-level random effects for the intercept and linear trajectory components and a provider-level random effect for the intercept. The initial model also included the number of therapy sessions and digital exercises completed during the past 7 days, as well as the amount of time spent engaged with digital lessons (in 10-minute intervals) as TVCs.

Next, in Model 2, we incorporated a dummy-coded variable indicating the phase of treatment (late phase: Weeks 0-7=0; Weeks 8-15=1) along with the interaction terms between the phase indicator and the engagement TVCs. This allowed the magnitude of TVC effects to differ across the early and late phases of treatment; i.e., the inclusion of the late phase*TVC interaction terms means that the first-order coefficients for therapy sessions, digital lessons, and exercises represent the simple effects of these predictors during the early phase of the study period [31]. In addition, the interaction terms describe the change in the magnitude of each TVC when considered during the late phase relative to the early phase.

In Model 3, several demographic variables were added as TICs to examine the impact of age, gender, and race on individual trajectories. Finally, several sensitivity analyses were conducted to evaluate the impact of receiving a therapy session and engaging with digital lessons during the same week, along with the isolated association between digital exercise engagement and weekly symptoms.

## Results

### Participant Characteristics

Table 1 includes the participants’ demographic and baseline characteristics for the entire sample and each of the analysis samples (GAD-7 and PHQ-9), as well as the engagement in therapy sessions, digital lessons, and exercises during the first 16 weeks.

**Figure 2:**
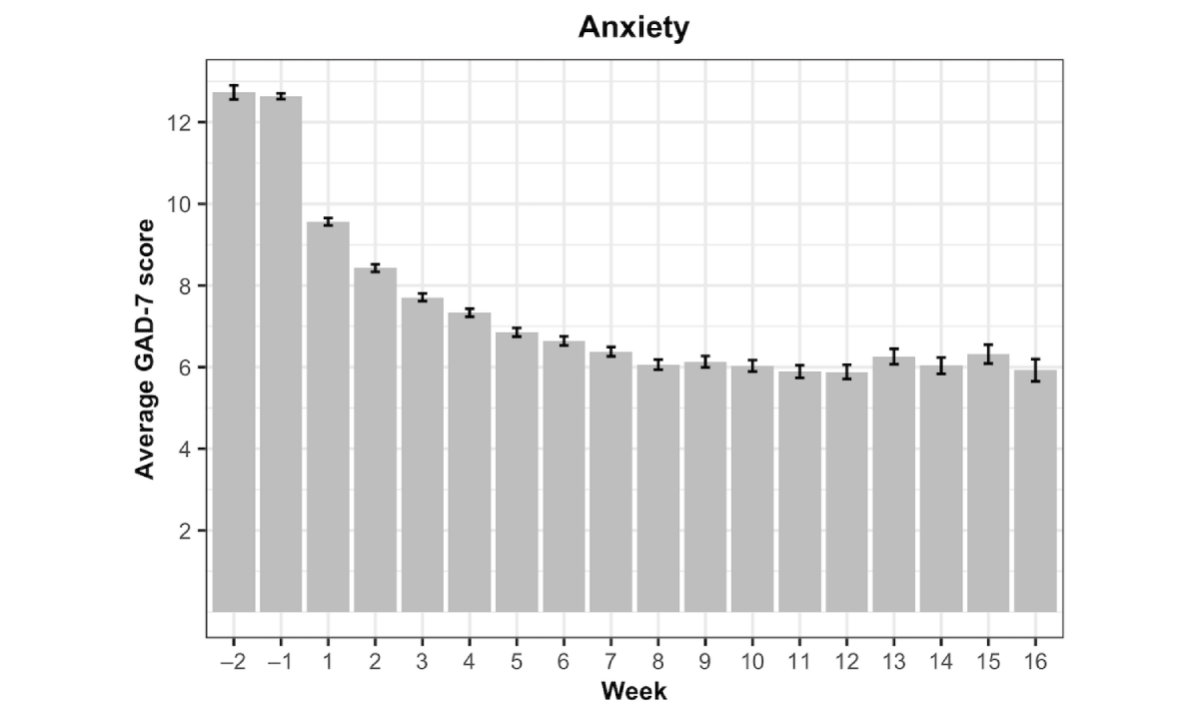
Mean Generalized Anxiety Disorder-7 (GAD-7) scores by week.

**Figure 3:**
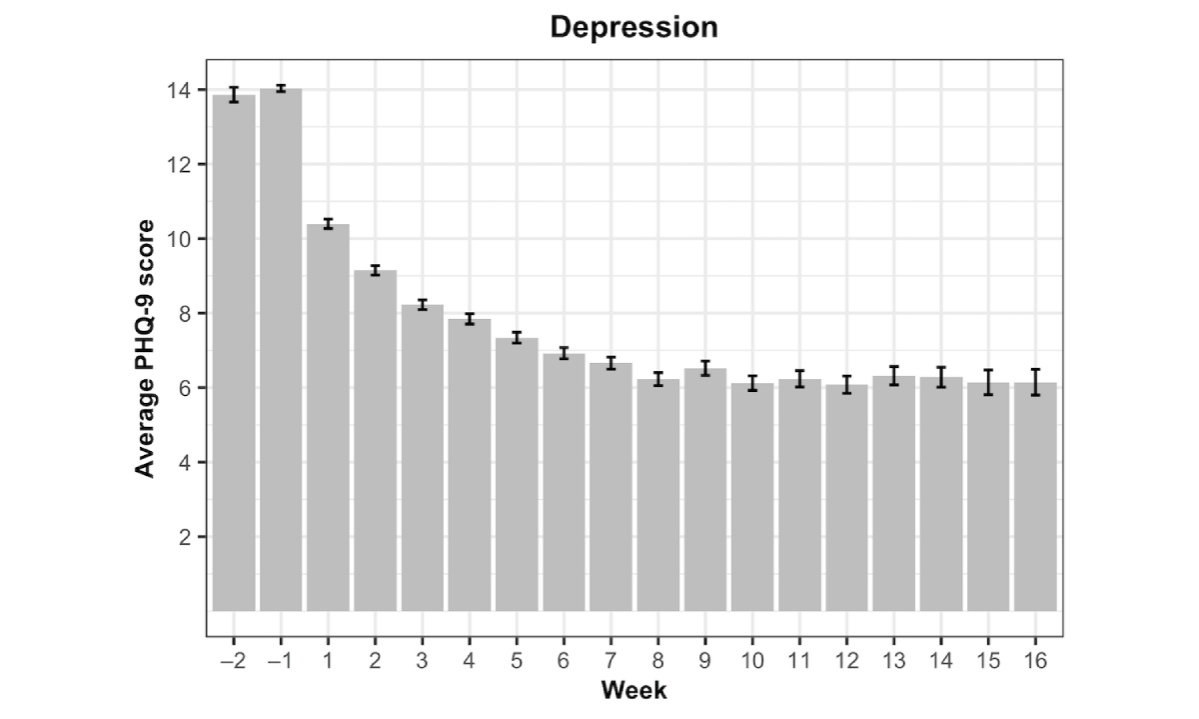
Mean Patient Health Questionnaire-9 (PHQ-9) scores by week.

Figures 2 and 3 are bar charts that respectively illustrate the GAD-7 and PHQ-9 mean scores over the study period. The length of the error bars represents the standard error of the mean. To interpret the “Week” values, Week=–1 is the week before the first session, and Week=1 is the first week after the first session.

**Table 1:** Demographic information and engagement in treatment components.

		Entire sample (N=3401)	GAD-7^a^ sample (n=3031)	PHQ-9^b^ sample (n=1999)
Age, mean (SD)		33.15 (8.68)	33.22 (8.66)	33.16 (8.82)
Female sex, n (%)		2218 (65.22)	1987 (65.56)	1304 (65.23)
**Race/ethnicity, n (%)**				
	Minority	1731 (50.90)	1547 (51.04)	1043 (52.18)
	Unknown	136 (4.00)	121 (3.99)	95 (4.75)
Baseline GAD-7 score, mean (SD)		11.83 (4.08)	12.64 (3.52)	12.14 (4.68)
Baseline PHQ-9 score, mean (SD)		10.72 (4.92)	10.49 (5.08)	13.99 (3.43)
Number of sessions completed, mean (SD)		5.82 (3.05)	5.84 (3.09)	5.97 (3.14)
**Total number of out-of-session engagements, mean (SD)**		43.36 (28.34)	43.39 (28.45)	44.80 (29.49)
	Number of lessons completed, mean (SD)	5.56 (3.54)	5.53 (3.52)	5.67 (3.65)
	Number of exercises completed, mean (SD)	10.56 (12.16)	10.49 (12.10)	10.96 (12.57)
Duration of care (weeks), mean (SD)		6.97 (4.71)	6.98 (4.72)	7.13 (4.79)

^a^GAD-7: Generalized Anxiety Disorder-7.

^b^PHQ-9: Patient Health Questionnaire-9.

### GAD-7 Score

Model 1 featured a linear and a quadratic trajectory and time-varying indicators of engagement (therapy sessions, digital lessons, and digital exercises). The fixed coefficient for the first-order effect of the treatment week revealed that on average, GAD-7 symptom scores decreased considerably during the first week of treatment (b=–1.09, 95% CI –1.12 to –1.06; *P*<.001). However, a significant quadratic coefficient (b=0.05, 95% CI 0.05-0.05; *P*<.001) suggests that the rate of decline diminishes over time. Fixed effect coefficients for the TVCs revealed that clients who attended a therapy session during the last 7 days reported significantly lower GAD-7 scores (b=–0.68, 95% CI –0.77 to –0.59; *P*<.001), and that every 10 minutes of digital lessons completed was associated with significantly lower GAD-7 scores (b=–0.14, 95% CI –0.19 to –0.08; *P*<.001). Contrary to our expectations, engagement with digital exercises was associated with a slight increase in GAD-7 scores (b=0.03, 95% CI 0.00-0.06; *P*=.045).

In Model 2, the TVC coefficients were allowed to differ across the phases of treatment. The first-order effect of the phase indicator variable (late phase) suggests that the average anxiety trajectory shifts upwards slightly during the later phase of treatment (b=0.44, 95% CI 0.20-0.68; *P*<.001), relative to the early phase. A significant late phase*therapy sessions interaction emerged (b=0.25, 95% CI 0.03-0.47; *P*=.03), suggesting that the beneficial effect of a session became weaker during the late phase of treatment. A similar pattern emerged for digital lessons, such that the beneficial effect of completing 10 minutes of lessons was significantly weaker (b=0.22, 95% CI 0.07-0.36; *P*=.004) during the late phase of treatment. In contrast, the phase interaction involving digital exercises failed to reach significance.

Model 3 contained the same configuration of TVCs and phase interactions as Model 2, but incorporated several client-level TICs examining the impact of demographic variables. Although no differences in initial GAD-7 scores emerged as a function of age or race, females reported slightly higher baseline anxiety scores (b=0.37, 95% CI 0.15-0.60; *P*=.001) relative to male clients. The coefficients describing TVC effects across early and late phases were identical to Model 2. In sum, anxiety symptoms declined significantly over the course of treatment overall. Additionally, engagement with weekly therapy sessions and digital lessons was associated with lower anxiety symptoms, particularly during the early phase of treatment. Table 2 includes all point estimates and 95% confidence intervals for fixed effects for the GAD-7 analyses, and Table 3 shows the deviance and selection criteria.

**Table 2:** Generalized Anxiety Disorder-7 analysis results (n=3031).

	Model 1				Model 2				Model 3		
	Estimate (95% CI)	*t*_obs_^a^ (*df*)	*P* value	Estimate (95% CI)		*t*_obs_ (*df*)	*P* value	Estimate (95% CI)		*t*_obs_ (*df*)	*P* value
Intercept	11.31 (11.17 to 11.46)	153.80 (297)	<.001	11.36 (11.22 to 11.51)		153.62 (307)	<.001	11.16 (10.93 to 11.40)		91.87 (1485)	<.001
Week	–1.09 (–1.12 to –1.06)	65.75 (15,000)	<.001	–1.09 (–1.13 to –1.06)		65.89 (15,000)	<.001	–1.09 (–1.13 to –1.06)		65.91 (15,000)	<.001
Week^2^	0.05 (0.05 to 0.05)	36.12 (16,000)	<.001	0.04 (0.04 to 0.05)		29.81 (17,000)	<.001	0.04 (0.04 to 0.05)		29.82 (17,000)	<.001
Therapy sessions in the last 7 days	–0.68 (–0.77 to –0.59)	14.36 (18,000)	<.001	–0.72 (–0.82 to –0.61)		13.58 (18,000)	<.001	–0.72 (–0.82 to –0.61)		13.59 (18,000)	<.001
Digital exercises in the last 7 days	0.03 (0.00 to 0.06)	2.01 (19,000)	.04	0.03 (0.00 to 0.06)		2.12 (19,000)	.03	0.03 (0.00 to 0.06)		2.10 (19,000)	.04
Digital lessons in the last 7 days	–0.14 (–0.19 to –0.08)	4.74 (18,000)	<.001	–0.15 (–0.21 to –0.09)		4.91 (18,000)	<.001	–0.15 (–0.21 to –0.09)		4.86 (18,000)	<.001
Late phase	—^b^	—	—	0.44 (0.20 to 0.68)		3.60 (18,000)	<.001	0.44 (0.20 to 0.68)		3.61 (18,000)	<.001
Late phase*therapy sessions in the last 7 days	—	—	—	0.25 (0.03 to 0.47)		2.19 (18,000)	.03	0.25 (0.03 to 0.47)		2.20 (18,000)	.03
Late phase*digital exercises in the last 7 days	—	—	—	0.04 (–0.03, to 0.11)		1.06 (18,000)	.29	0.04 (–0.03 to 0.11)		1.07 (18,000)	.29
Late phase*digital lessons in the last 7 days	—	—	—	0.22 (0.07 to 0.36)		2.88 (18,000)	.004	0.21 (0.07 to 0.36)		2.85 (18,000)	.004
Age	—	—	—	—		—	—	0.00 (–0.01 to 0.01)		0.27 (2941)	.79
Gender	—	—	—	—		—	—	0.37 (0.15 to 0.60)		3.24 (2986)	.001
Minority ethnicity	—	—	—	—		—	—	–0.13 (–0.35 to 0.09)		1.15 (2984)	.25
No ethnicity reported	—	—	—	—		—	—	0.47 (–0.09 to 1.02)		1.64 (3016)	.10

^a^*t*_obs_: observed *t* test result.

^b^—: not applicable (variables not entered into model).

**Table 3:** Deviance and selection criteria for the 3 models in the Generalized Anxiety Disorder-7 analysis.

	Model 1	Model 2	Model 3
Deviance (–2LL^a^)	111676.4	111622.0	111620.8
Akaike information criterion	111686.4	111632.0	111630.8
Bayesian information criterion	111704.2	111649.9	111648.7

^a^—2LL: log-likelihood ratio.

### PHQ-9 Score

As before, the fixed coefficient for the first-order effect of treatment week for Model 1 revealed that on average, PHQ-9 scores decreased significantly during the first week of treatment (b=–1.24, 95% CI –1.28 to –1.20; *P*<.001). A significant quadratic coefficient (b=0.05, 95% CI 0.05-0.06, *P*<.001) indicates that the rate of decline diminishes over the course of treatment. Fixed effect coefficients for the TVCs revealed that clients who attended a therapy session during the last 7 days reported significantly lower PHQ-9 scores (b=–0.73, 95% CI –0.85 to –0.61; *P*<.001). In addition, completion of digital exercises (b=–0.04, 95% CI –0.08 to 0.00; *P*=.03) and digital lessons (b=–0.01, 95% CI –0.17 to –0.03; *P*=.008) over the prior week was associated with significantly lower PHQ-9 scores.

In Model 2, the first-order effect of late phase suggests that the average depression trajectory shifts upwards slightly during Weeks 8 to 15 of treatment (b=0.38, 95% CI 0.08-0.69; *P*=.01). A significant late phase*therapy sessions interaction emerged (b=0.56, 95% CI 0.27-0.84; *P*<.001), suggesting that the beneficial effect of a session became weaker during the late phase of treatment. A similar pattern emerged for digital lessons, such that the beneficial effect of completing 10 minutes of lessons was significantly weaker (b=0.28, 95% CI 0.09-0.46; *P*=.003) during the late phase of treatment. In contrast, the phase interaction involving digital exercises failed to reach significance.

For Model 3, no differences in initial PHQ-9 scores emerged as a function of age, race, or gender. Overall, depression symptoms declined significantly over the course of treatment. Additionally, engagement with all program elements was associated with lower depression symptoms, with therapy sessions and digital lessons exhibiting a stronger effect during the early phase of treatment. Table 4 includes all point estimates and 95% confidence intervals for fixed effects for the PHQ-9 analyses, and Table 5 shows the deviance and selection criteria.

**Table 4:** Patient Health Questionnaire-9 analysis results (n=1999).

	Model 1			Model 2			Model 3		
	Estimate (95% CI)	*t*_obs_^a^ (*df*)	*P* value	Estimate (95% CI)	*t*_obs_ (*df*)	*P* value	Estimate (95% CI)	*t*_obs_ (*df*)	*P* value
Intercept	12.43 (12.24 to 12.62)	130.55 (276)	<.001	12.51 (12.32 to 12.70)	130.54 (284)	<.001	12.38 (12.06 to 12.71)	74.43 (1247)	<.001
Week	–1.24 (–1.28 to –1.20)	56.79 (9682)	<.001	–1.24 (–1.28 to –1.20)	56.89 (9714)	<.001	–1.24 (–1.28 to –1.20)	56.88 (9717)	<.001
Week^2^	0.05 (0.05 to 0.06)	30.74 (11,000)	<.001	0.05 (0.04 to 0.05)	25.51 (12,000)	<.001	0.05 (0.04 to 0.05)	25.51 (12,000)	<.001
Therapy sessions in the last 7 days	–0.73 (–0.85 to –0.61)	11.87 (12,000)	<.001	–0.82 (–0.96 to –0.69)	12.07 (12,000)	<.001	–0.83 (–0.96 to –0.69)	12.08 (12,000)	<.001
Digital exercises in the last 7 days	–0.04 (–0.08 to 0.00)	2.18 (13,000)	.03	–0.03 (–0.07 to 0.01)	1.60 (13,000)	.11	–0.03 (–0.07 to 0.01)	1.59 (13,000)	.11
Digital lessons in the last 7 days	–0.10 (–0.17 to –0.03)	2.67 (12,000)	.01	–0.12 (–0.19 to –0.04)	2.95 (12,000)	.003	–0.12 (–0.19 to –0.04)	2.94 (12,000)	.003
Late phase	—^b^	—	—	0.38 (0.08 to 0.69)	2.45 (12,000)	.01	0.38 (0.08 to 0.69)	2.45 (12,000)	.01
Late phase*therapy sessions in the last 7 days	—	—	—	0.56 (0.27 to 0.84)	3.82 (12,000)	<.001	0.56 (0.27 to 0.84)	3.83 (12,000)	<.001
Late phase*digital exercises in the last 7 days	—	—	—	0.02 (–0.07 to 0.11)	0.43 (12,000)	.67	0.02 (–0.07 to 0.11)	0.43 (12,000)	.67
Late phase*digital lessons in the last 7 days	—	—	—	0.28 (0.09 to 0.46)	2.96 (12,000)	.003	0.28 (0.09 to 0.46)	2.96 (12,000)	.003
Age	—	—	—	—	—	—	0.01 (–0.01 to 0.02)	0.61 (1986)	.54
Gender	—	—	—	—	—	—	0.23 (–0.09 to 0.54)	1.41 (1991)	.16
Minority ethnicity	—	—	—	—	—	—	–0.07 (–0.38 to 0.24)	0.43 (1998)	.67
No ethnicity reported	—	—	—	—	—	—	0.28 (–0.45 to 1.00)	0.75 (2058)	.45

^a^*t*_obs_: observed *t* test result.

^b^—: not applicable (variables not entered into model).

**Table 5:** Deviance and selection criteria for the 3 models in the Patient Health Questionnaire-9 analysis.

	Model 1	Model 2	Model 3
Deviance (–2LL^a^)	76960.5	76905.2	76913.4
Akaike information criterion	76970.5	76915.2	76923.4
Bayesian information criterion	76988.2	76932.8	76941.1

^a^—2LL: log-likelihood ratio.

### Sensitivity Analysis

To investigate the extent to which the unexpected findings for the digital exercises in Model 1 of the GAD-7 analysis can be explained by multicollinearity or suppression effects, we specified a series of follow-up models examining digital exercises in isolation. A significant effect emerged, such that completing a digital exercise was associated with an expected b=–0.05 unit decrease in GAD-7 scores (95% CI –0.08 to –0.02; *P*<.001).

## Discussion

Findings from this study revealed significant declines in anxiety and depression symptoms, with a steep initial decline that became less pronounced over time. The initial model showed that engagement with therapy sessions and digital lessons during the prior 7 days was uniquely associated with lower anxiety and depression symptoms. Although engagement with digital exercises over the past week was associated with lower depression symptoms, a positive coefficient emerged in the analysis on anxiety symptoms. Sensitivity analyses revealed that the unexpected positive relationship between use of digital exercises and anxiety symptoms may be attributable to collinearity with therapy sessions and digital lessons. Analyses examining differences across the early (Weeks 0-7) and late (Weeks 8-15) phases of therapy found that for both anxiety and depression, therapy sessions and digital lessons (but not digital exercises) were significantly stronger (more negative) during the early phase of treatment. These effects remained after incorporating age, gender, and ethnicity.

Overall, significant declines in both anxiety and depression were observed throughout treatment, demonstrating the efficacy of BC-CBT in decreasing these symptomatologies. These robust effects lend support to a blended care model in addressing anxiety and depressive symptoms [16], and such a model can be a potentially more scalable and cost-effective method of providing evidence-based care compared to traditional methods of delivering psychotherapy [15]. Additionally, steeper declines in symptom severity were observed during the early stages of therapy, and therapy sessions and video lessons were significantly more potent in decreasing symptomatology during the early phase of treatment. Given that the initial stages of therapy are largely dedicated to providing pertinent psychoeducation and establishing key therapeutic concepts and skills, therapy sessions and digital video lessons appear to be effective ways to reinforce the uptake of this crucial information. Specifically, a key component of early psychoeducation is to establish the rationale for treatment and clarify how current cognitive and behavioral patterns contribute to the maintenance of symptomology. This crucial information has been shown to facilitate client reappraisals and contributes to lower avoidance of challenging situations early on in treatment [32], which is key for decreasing symptom severity and functional impairment. Consequently, providers should capitalize on the robust effects of these treatment components earlier on in therapy, ensuring that sessions are occurring consistently and that video lessons are being routinely assigned to clients in order to reinforce key therapeutic concepts. These findings also highlight the importance of setting positive expectations and obtaining client buy-in early on in therapy [33], given that the early phase of treatment often incites hope and a sense of agency [34]. This will optimally position clients to be their own enactors of change, motivating them to learn and practice coping skills later on in therapy.

Indeed, the later phase of therapy is primarily dedicated to the actual practice of skills in day-to-day life, which is challenging for clients as they start to practice these coping skills in more difficult, real-world situations. As such, it is not surprising to see a slower decline in symptoms in the latter stage of therapy, which is expected due to the increased difficulty of therapeutic tasks [17,35]. The later stages of therapy are critical to achieving reliable clinical improvement and durability of treatment gains, given the opportunities for real-world generalization of therapy skills. To facilitate these crucial steps, future studies would benefit from investigating how to increase the potency of digital tools and engagement in the later phase and further develop digital content that is better suited for later stages in treatment (eg, more in-depth skills review and practices). Ultimately, although the early and late phases of therapy have unique focuses, they both play crucial roles in achieving positive treatment outcomes.

Having a therapy session and watching digital video lessons were uniquely associated with decreases in anxiety and depression, highlighting the potency of both traditional face-to-face therapy and the digital components within a blended care model. Meeting with a therapist for regular sessions allows the client to receive individualized care, in-depth exploration of key concepts and skills, and increased accountability. Indeed, therapy sessions were relatively more impactful in decreasing symptomology, underscoring the importance of therapist-led treatment [36,37]. It is notable that completing digital video lessons also uniquely contributed to decreases in anxiety and depression, supporting its potential standalone utility. The digital video lessons help teach and reinforce key therapy concepts in an engaging manner, allow for asynchronous review of the materials at the client’s convenience, and facilitate dissemination of therapeutic content at scale. These benefits are particularly important to consider when resources are limited and therapy sessions cannot be conducted with regularity, which can be affected by financial barriers, difficulties obtaining reliable transportation to/from sessions, or lack of access to trained professionals [38,39]. In these cases, digital video lessons can be pivotal in expanding access to evidence-based treatment, given the support that digital interventions have received for effectively reducing access barriers [40].

There was an unexpected finding of digital exercises being associated with increased anxiety symptoms, but the effect size was relatively weak. Based on the sensitivity analyses, the follow-up models indicated a potential suppression effect for digital exercises; digital exercises in isolation contributed to decreases in anxiety and depressive symptoms. These findings are more consistent with extant literature, which reports a strong association between greater homework compliance and better treatment outcomes [41,42]. The relatively small effect sizes observed can be attributed to the measurement method of exercise completion. In fact, previous research has reported smaller effect sizes when homework completion is measured objectively and contemporaneously [41], both of which are typical with digital platforms. Additionally, clients may have generalized the skills to day-to-day life as therapy progressed and gradually decreased reliance on the digital exercise [21], causing them to practice the skills taught in the exercises but not actually log them in the platform, thereby diluting the strength of the association. Future studies should seek to further disentangle the overlap between various digital components of care and better establish the unique contributions through randomly assigned conditions and/or sequencing studies.

These findings should be considered within the context of several limitations and suggested future directions for research. First, this study employs a pragmatic retrospective design, limiting the ability to rule out regression to the mean effects. Future studies should seek to examine the efficacy of BC-CBT and its care components in a randomized controlled trial to instill further confidence in this model beyond a control condition. Within these limitations, it is important to note that the naturalistic design in real-world settings strengthens the ecological validity, and the growth curve model allows us to glean nuanced information about the temporality of changes and account for stable and varying client-level differences. Second, self-report measures were used to determine clinical outcomes. Although the measures used are extensively validated and possess strong psychometric properties, future studies should use a multimethod, multi-informant approach to measuring symptomatology to establish a more comprehensive evaluation [43]. Third, more general therapy process variables were not incorporated into the analyses. As such, future studies should seek to better understand the impact of these factors (eg, therapeutic alliance, treatment expectations, familiarity with technology, therapist reinforcement of skills practice) on engagement with digital activities and treatment outcome [44]. Fourth, the measurement of digital exercise completion could be improved in future studies. Although real-time data were gathered whenever clients completed exercises in the platform, data collection is incumbent upon the client electronically logging it, so it is possible that clients were practicing the coping skills noted in the digital exercise but not actually logging it in the system. Additionally, more nuanced data regarding the actual time clients spent on digital exercises may be helpful to examine in relation to outcomes. Fifth, given that the number of exercises assigned decreases significantly in the later phase of therapy, it is possible that there was less power to detect the impact of exercises on decreasing symptomology. Sixth, given that other types of digital engagement occurred in between sessions (eg, completion of assessments, messages between provider and client), it would also be helpful to examine their impact on treatment outcomes. Although they are inherently different from digital therapy content (ie, digital content directly teaching therapeutic concepts and skills), these other types of engagement may uniquely impact engagement in therapy overall. Seventh, this study focused on treatment outcomes during the acute phase of therapy. Future studies should seek to conduct long-term follow-ups to determine how robust BC-CBT is in maintaining treatment gains for anxiety and depression. Eighth, although anxiety and depression were the primary focuses of the study, clients may have had other presenting problems as well. Future studies should seek to examine the impact of comorbidities on treatment outcome. Ninth, this study focused on distinct outcomes (ie, anxiety and depression symptoms), and many clients met inclusion criteria for both conditions, resulting in their inclusion in both sets of analyses. Although this does not pose a meaningful threat to the validity of the analyses presented here, future studies drawing on larger samples should explore whether clients with comorbid presentations respond differently to the BC-CBT intervention than clients who only meet criteria for one condition. Tenth, providers anchored their interventions in CBT-based approaches and were able to implement a variety of interventions (eg, DBT, ACT). It would be of interest in future studies to investigate the potential impact of different approaches on outcomes.

Collectively, these findings highlight the robust effectiveness of a BC-CBT program in decreasing anxiety and depression in real-world settings. Given the myriad barriers to accessing evidence-based care, blended care programs combine the advantages of technology with traditional face-to-face therapy to facilitate greater dissemination of CBT. Timing of treatment components and the potency of certain digital activities (ie, video lessons) should be considered when implementing a blended care model. Ultimately, the unique contributions of various treatment components underscore the utility of a blended care model in effectively treating depression and anxiety.

## Abbreviations

ACT: acceptance and commitment therapy
BC-CBT: blended care cognitive behavioral therapy
CBT: cognitive behavioral therapy
DBT: dialectical behavior therapy
GAD-7: Generalized Anxiety Disorder-7
HIPAA: Health Insurance Portability and Accountability Act
iCBT: internet-based cognitive behavioral therapy
MAR: missing at random
PHQ-9: Patient Health Questionnaire-9
TIC: time-invariant covariate
TVC: time-varying covariate
